# Management of acute appendicitis in pregnancy (MAMA): Protocol for a multicentre observational study

**DOI:** 10.1371/journal.pone.0330502

**Published:** 2025-08-14

**Authors:** Nilofer Husnoo, Ian Henderson, Hajra Khattak, Benjamin Rea, Catriona Wright, Brenda Narice, Jack Helliwell, Adil Rashid, Babu Karavadra, Sonia Lockwood, Arin Saha, Stephen Gerry, Stephen J. Chapman

**Affiliations:** 1 Department of Clinical Medicine, School of Medicine and Population Health, University of Sheffield, Sheffield, United Kingdom; 2 Department of General Surgery, Sheffield Teaching Hospitals NHS Foundation Trust, Sheffield, United Kingdom; 3 National Perinatal Epidemiology Unit, Nuffield Department of Population Health, University of Oxford, Oxford, United Kingdom; 4 Department of Reproductive Health, Elizabeth Garrett Anderson Institute for Women’s Health, University College London, London, United Kingdom; 5 Department of Radiology, Sheffield Teaching Hospitals NHS Foundation Trust, Sheffield, United Kingdom; 6 Leeds Institute of Medical Research, University of Leeds, Leeds, United Kingdom; 7 Institute of Population Health, University of Liverpool, Liverpool, United Kingdom; 8 Department of General Surgery, Bradford Teaching Hospitals NHS Foundation Trust, Bradford, United Kingdom; 9 Department of General Surgery, Calderdale and Huddersfield NHS Foundation Trust, Huddersfield, United Kingdom; 10 Nuffield Department of Orthopaedics, Centre for Statistics in Medicine, Rheumatology and Musculoskeletal Sciences, University of Oxford, Oxford, United Kingdom; Imperial College London, UNITED KINGDOM OF GREAT BRITAIN AND NORTHERN IRELAND

## Abstract

**Introduction:**

Acute appendicitis is the most common emergency general surgical condition in pregnancy, affecting around 1 in 1000 pregnancies. Reaching a diagnosis is more challenging in this group because of altered clinical presentations and the limitations of diagnostic tools in pregnancy. There is limited guidance from national bodies in the UK on the management of this condition. This study aims to describe current diagnostic and treatment practices for acute appendicitis in pregnancy in the UK, and associated outcomes, to identify variations in practice and areas for improvement.

**Methods and analysis:**

This is a UK-wide observational study. Hospitals providing an emergency general surgical service will be eligible to participate. The study consists of two components: (1) a site resource-profile questionnaire to assess the setup of services to care for the pregnant patient requiring emergency non-obstetric abdominal surgery to be completed by a senior collaborator at each participating site, and (2) a retrospective study of patients diagnosed with appendicitis or who had an appendicectomy for suspected appendicitis during a ten-year study period (2013–2023). Data will be collected on diagnostics, radiological findings, management approach and surgical and obstetric outcomes. The main measure of interest is the management approaches (operative versus non-operative; laparoscopic versus open surgery) and their association with selected outcomes. Multivariable logistic regression analyses will be conducted to identify factors which might predict the management strategy and outcomes.

**Ethics and dissemination:**

The protocol for this service evaluation study has been reviewed by the University of Sheffield Research Ethics Committee. The study findings will be discussed in a multistakeholder workshop consisting of general surgeons, obstetricians and gynaecologists, radiologists, anaesthetists and patient representatives with a view to making recommendations for quality improvement work and further research. Key findings and recommendations will be disseminated through specialist societies.

## Introduction

Acute appendicitis is the most common emergency general surgical condition in pregnancy [1], affecting 1 in 1000 pregnancies [2,3]. The timely diagnosis of appendicitis in this cohort is essential owing to the increased risk of maternal and foetal morbidity associated with delayed treatment. Indeed, the rate of pregnancy loss increases in complex disease (such as perforated or necrotic appendicitis) (10–35%) compared to simple disease (<5%) [4–7]. Reaching an accurate clinical or radiological diagnosis in a timely fashion, however, remains a challenge in the pregnant patient.

The classical presentation of right lower quadrant pain is less common in pregnancy [8]. The gravid uterus tends to displace the appendix superiorly [9]. This makes it challenging to recognise appendicitis clinically, particularly due to the wide range of differential diagnoses which are relevant to the pregnant patient, such as cholecystitis, urinary tract infection, round ligament pain, miscarriage, amongst others [10]. Biochemical markers, such as leucocytosis, are less helpful in making a diagnosis in pregnant patients as mild leucocytosis is a common observation in pregnancy [11].

The accuracy of imaging modalities is also affected by the gravid uterus. Ultrasound scanning (US) is often the first modality of choice for diagnosing acute appendicitis in the pregnant patient. However, the accuracy of US in this context is low, with an overall sensitivity and specificity of 77.6% (95% CI, 75.4–79.6%) and 75.3% (95% CI, 73.1–77.5%), falling steadily from the first to the third trimester [12]. Magnetic resonance imaging (MRI) without gadolinium is the next preferred modality. It has been shown to be highly sensitive and specific, although the rate of non-visualisation of the appendix is higher in the 3^rd^ trimester [13]. MRI can be considered safe in pregnancy based on current recommendations [14,15]. However, it is not known whether MRI scanning is readily accessible across various centres in the UK. Computed tomography (CT) is the modality of choice for diagnosing appendicitis in the non-pregnant patient and is widely available in most healthcare systems. However, its use during pregnancy is discouraged due to the risk of ionising radiation and potential adverse foetal effects.

A number of national and international guidelines for the treatment of appendicitis in pregnancy have been produced (Table 1). There is consensus that operative management (appendicectomy), should be the preferred approach, since this is associated with lower maternal and foetal morbidity compared to non-operative management [2,16]. Guidelines are less consistent, however, in their recommendations on the best operative approach in the pregnant patient (laparoscopic or open). The World Society of Emergency Surgery guidelines favour laparoscopic appendicectomy [17], but European and American guidelines recommend laparoscopy only when the uterine fundus is below the level of the umbilicus [18,19]. Guidelines in the UK do not favour one over the other [20]. This lack of consensus may be explained by the fact that some studies have suggested an increased risk of foetal loss with laparoscopic surgery [5,21].

**Table 1:** Summary of recommendations for the treatment of acute appendicitis in pregnancy.

Guidelines	Year of publication	Operative vs non-operative management	Laparoscopic vs open appendicectomy
Evidence-Based Guideline on Laparoscopy in Pregnancy: Commissioned by the BSGE, endorsed by the RCOG [20]	2019	Recommend operative over non-operative management.	Do not recommend one approach over the other. Recommend that laparoscopic appendicectomy should only be performed by experienced laparoscopic surgeons.
Guidelines on general surgical emergencies in pregnancy [22]	2024	Recommend operative over non-operative management.	No recommendation on the best approach has been made in these guidelines.
EAES rapid guideline: systematic review, meta-analysis, GRADE assessment, and evidence-informed European recommendations on appendicitis in pregnancy [18]	2022	Strong recommendation for operative over non-operative management in complicated appendicitis. Weak recommendation of operative over non-operative management for uncomplicated appendicitis and no appendicolith on imaging.	Laparoscopic appendicectomy recommended until the 20th week of gestation, or when the fundus of the uterus is below the level of the umbilicus. Suggest laparoscopic or open appendicectomy beyond this, depending on the expertise of the surgeon.
SAGES guidelines for the use of laparoscopy during pregnancy [19]	2024 (update of 2017 guidance [23])	Recommend operative over non-operative management.	Laparoscopic appendicectomy recommended when the fundus of the uterus is below the level of the umbilicus. Suggest laparoscopic or open appendicectomy beyond this, depending on the expertise of the surgeon.
Diagnosis and treatment of acute appendicitis: 2020 update of the WSES Jerusalem guidelines [17]	2020	Recommend operative over non-operative management.	Recommend laparoscopic over open appendicectomy (where laparoscopic expertise is available).

BSGE: British Society for Gynaecological Endoscopy; EAES: European Association of Endoscopic Surgery; GRADE: Grading of Recommendations, Assessment, Development, and Evaluations; RCOG: Royal College of Obstetricians and Gynaecology; SAGES: Society of American Gastrointestinal and Endoscopic Surgeons; WSES: World Society of Emergency Surgery.

Little is known about current diagnostic and treatment practices for acute appendicitis in pregnancy in the UK. The current evidence is limited by numerous methodological challenges as well as the relative low frequency of appendicitis in pregnancy leading to an abundance of single centre and poorly generalisable reports. Real-world evidence on current practices and areas of variation is essential to guide future quality improvement initiatives and research for this important group of patients. The Management of Appendicitis in pregnancy (MAMA) study aims to address this issue by conducting a national, trainee-led collaborative assessment of current practices in the UK.

## Aims and objectives

The aim of this study is to understand the management of acute appendicitis in pregnancy in the UK and to identify possible variation in practice.

The objectives are to explore the characteristics of women who present with acute appendicitis during pregnancy, describe the approach to clinical investigation and the management of patients with this condition, and finally, to describe and compare maternal and perinatal process measures and outcomes according to the management group (non-operative versus operative and laparoscopic versus open).

In doing so, the study will describe the UK experience of non-operative and operative management, and where applicable, the experience of laparoscopic and open surgical approaches, and identify risk factors for non-operative management and for composite complication.

## Methods

### Summary of design

A national, retrospective, observational assessment of the management of acute appendicitis in pregnancy across the UK will be performed. Each participating centre will complete a hospital-level resource profile questionnaire to describe access to diagnostic services and the organisation of cross-specialty care. Data collection for the observational assessment of practice will take place across a 10 year period between 1st October 2013 and 30th September 2023 using routinely collected data.

### Ethics & data governance

The Health Research Authority (HRA) reviewed and confirmed that HRA and NHS Research Ethics Committee approval was not required for this study to take place in NHS hospitals. The study was prospectively approved by the University of Sheffield Research Ethics Committee (ref 056754) on 28^th^ November 2023. To facilitate the study at local sites, investigators will register the study according to local Research & Development protocols and seek Caldicott Guardian approval for the transfer of anonymised patient data. Patients who have opted out of routine data collection via the NHS National Data opt-out service will not be included. The researchers/ authors of this study will only have access to fully anonymised data.

### Site resource profile questionnaire

A site-specific questionnaire will be completed once by the lead investigator at each participating site (Table 1 in S1 Appendix). The purpose of this questionnaire is to describe:

1. The resources and imaging modalities available in- and out-of-hours to manage acute appendicitis in pregnancy
2. The existence of local protocols and treatment pathways for the management of acute appendicitis in pregnancy
3. The organisation of cross-specialty care within each trust

The data from this questionnaire will provide context for the findings from the observational study. The questionnaire will be administered using Qualtrics (https://www.qualtrics.com/), a secure online platform enabling investigators to complete the questionnaire on a computer or mobile device.

### Study setting

Any National Health Service (NHS) hospital in the UK offering an emergency general surgical service will be eligible to take part. It is therefore not possible to provide a list of participating centres at this stage; this will only be available when site recruitment is complete. A team of up to three investigators will facilitate the study at each hospital, comprising one resident doctor in General Surgery, one resident doctor in Obstetrics and Gynaecology, and one consultant in either General Surgery or Obstetrics and Gynaecology). The study will be coordinated by the White Rose Surgical Collaborative and the UK Audit and Research Collaborative in Obstetrics and Gynaecology.

### Eligibility criteria

Patients will be identified from a review of hospital-specific clinical coding (International Classification of Diseases diagnostic codes – ICD-10 – and Operating Procedure Codes Supplement – OPCS – Classification of Interventions and Procedures) between 1st October 2013 and 30th September 2023. The search strategy was devised with input from the coding team in a large tertiary unit. The approach was then used in the pilot phase and generated the expected number of cases. Previous UK-based studies on the risk of acute appendicitis in pregnancy, and outcomes of non-obstetric surgery in pregnancy, also used ICD-10 and OPCS codes [24,25]. Patients aged 18 years and above with a clinical, radiological, operative or histological diagnosis of acute appendicitis during any stage of a confirmed pregnancy will be eligible. Patients were managed non-operatively in the setting of equivocal radiological assessments will still be included provided that a definitive clinical diagnosis of acute appendicitis was made by a surgical specialist.

### Outcomes & Measures

The primary measure of interest is management approach to acute appendicitis (non-surgical versus surgical, and laparoscopic versus open). The following clinical outcomes and process measures will also be assessed:

1. Time interval from initial presentation to imaging
2. Incidence of complicated appendicitis (perforation or abscess formation)
3. Total length of hospital stay (days)
4. Incidence of preterm birth (<37 weeks of completed gestation)
5. Small for gestational age (Intergrowth-21^st^ international standard [26])
6. Composite 30-day medical, surgical and obstetric complication from the index presentation. Composite complications will comprise wound infection, intra-abdominal collection, prolonged ileus, hospital-acquired pneumonia, venous thromboembolism, return to theatre, admission to ITU or critical care, death; pregnancy loss
7. Incidence of unplanned readmission within 30 days
8. Recurrence of acute appendicitis during pregnancy

### Data collection

Routinely available data will be extracted from free-text clinical notation, laboratory reports, investigation and histopathological reports, operative reports, and the maternal record from both electronic and paper records. Although most centres in the UK now have an electronic patient record system, paper records are still generally retrievable, which became evident during the early stages of the study. Anonymised data will be entered securely into a REDCap (Research Electronic Data Capture) database, hosted by the University of Sheffield using a piloted data collection tool (Table 2 in S1 Appendix); no patient-identifiable data will be entered. Data on the exact year of diagnosis will not be collected to minimise the risk of patient identification; instead, data on the time period during which the diagnosis was made will be collected (i.e., former half of the study period vs. latter half). All electronic data will be held for up to five years, after which it will be permanently and safely removed according to local governance processes. Site recruitment opened in January 2024, and will remain open until at least February 2025; data collection will continue until June/July 2025. Patient records will be accessed for data collection purposes at individual sites once local approvals have been secured and will be allowed to continue until June/July 2025.

### Sample size

Using an estimate of 670,000 births annually in the UK [27–30] and a rate of 1 case per 1000, the ten year population is estimated to be in excess of 6700 cases of appendicitis. Data from three initial pilot sites yielded between 10 and 25 cases over the ten year study period. Given that cases will be sampled at the level of the hospital, an assumed intraclass correlation coefficient of 0.025 was used. Based on the pilot data, a target of at least 47 hospitals and 470 cases is set to achieve a 5% margin of error for a proportion of 0.5; that is, a 5% margin of error should the prevalence of surgical management be 50%. The margin of error for less balanced measures would therefore more precise. Additionally, based on the pilot data, reasons for potential missing cases will be collected to understand any potential differences between the rate of acute appendicitis based on administrative data compared with this case-based approach. However, there will be no limit on the maximum number of sites that can be part of the study.

Demographic and characteristic data will be described using counts, proportions, and averages (means with standard deviations; or medians and interquartile ranges, as appropriate), along with corresponding 95% confidence intervals. Process and clinical measures will be compared between management strategies (operative vs. non-operative; laparoscopic vs. open surgery) using linear or log-binomial regression models to identify how these approaches and associated outcomes differ. When comparing management strategies, adjustment will be made for patient characteristics that may confound the relationship between management strategy and measure/outcome (age, body mass index, pre-existing comorbidities, and gestational age). Time period of diagnosis will also be adjusted for. Multivariable regression models will be used to identify risk factors (such as age, body mass index, pre-existing comorbidities, radiological findings and gestational age) for non-operative management and composite complications; multilevel modelling will be used if appropriate to allow differences across centres to be taken into account. All analyses will use clustered standard errors. The quality of submitted data will be reviewed prospectively and any queries highlighted with the submitting team to ensure data quality. Analyses will be conducted using the latest version of Stata (StataCorp LLC, College Station, Texas). The statistical analysis plan was reviewed by an independent senior statistician.

### Patient and public involvement

A patient, who was previously treated for acute appendicitis whilst pregnant, was invited to comment on the study design and objectives and considered this to be important work with potential to lead to improved and more standardised care. A patient panel (consisting of patients with experience of this condition) is being set up to contribute to the interpretation and dissemination of results, as described below.

### Dissemination

The study findings will be discussed in a workshop consisting of relevant stakeholders (general surgeons, obstetricians and gynaecologists, radiologists, anaesthetists and patient representatives). Recommendations for quality improvement work and further research will be made. Findings will be presented at national and international meetings and will be published in peer-reviewed papers, and will be disseminated through specialist societies.

## Discussion

The management of acute appendicitis in the pregnant patient has multiple challenges and areas of uncertainty. Each general surgeon may only encounter this condition a few times throughout their career, making it difficult to build on individual experience. It is therefore important to have clear practice recommendations. To our knowledge, the MAMA study will be the first UK-based study to provide an evaluation of national diagnostic and management practices relating to acute appendicitis in pregnancy, offering insight into variations in practice and deviations from current recommendations. The findings have the potential to inform the development of clinical guidelines and quality indicators for the management of this condition.

The current evidence around this area of surgical and obstetric practice is limited. In the only other UK-wide study performed in recent times the authors reported the outcomes of non-obstetric surgery in pregnancy, using Hospital Episodes Statistics (HES) data spanning from 2002–2012 [24]. An increased risk of adverse birth outcomes in patients undergoing abdominal surgery was reported, with laparoscopic surgery contributing mostly to this risk. However, this study reported the outcomes of abdominal surgery in general, providing limited data on the cohort who underwent an appendicectomy. The reliance on administrative data limits the understanding of the diagnostic and treatment pathways. Our study aims to address this by gathering information from medical records, including timelines for imaging, investigative findings, and outcomes of both surgical and non-surgical approaches.

The national and collaborative scope of the MAMA study is considered to be a key strength. It will provide relevant evidence around the management of this important group via a rapid initiative capable of informing quality improvement work and identifying research needs. Limitations of the study are also recognised. The MAMA study is a retrospective assessment of practice based on routinely collected clinical data, with the inherent biases that accompany retrospective data collection. However, prospective studies are difficult to conduct in this setting due to the relatively low incidence of the condition. Monitoring of the quality of submitted data with real-time trouble shooting will reduce the risk that this poses. The hospital-level questionnaire will also inform the interpretation of the data from individual hospitals. As a national study, the MAMA study is expected to have a large sample size, making meaningful contribution to the current evidence-base, which mostly comprises smaller, single-centre studies.

The management of emergency general surgical conditions in pregnant patients is marked by considerable uncertainty. We do not know how acute appendicitis in pregnancy is managed in the UK. There is a clear need to describe the care of this cohort and to understand how variation in care may impact clinical outcomes. We anticipate that our study will establish a multidisciplinary network of clinicians united by a common interest in managing pregnant patients with acute intra-abdominal surgical conditions. This network will be positioned to make recommendations for quality improvement initiatives, prioritise future research directions, and facilitate the development of additional studies informed by the findings of the MAMA study.

## Supporting information

S1 Appendix Supplementary material. (DOCX)
