# Supplementary material for: Management of acute appendicitis in pregnancy (MAMA): Protocol for a multicentre observational study
Source: PLoS One. 2025 Aug 14;20(8):e0330502. doi: 10.1371/journal.pone.0330502 (PMC12352775; doi:10.1371/journal.pone.0330502)
Supplement: S1 Appendix — (DOCX) [file pone.0330502.s001.docx]

| \| **Table 1: Site resource profile questionnaire** \| \| \| \| \| --- \| --- \| --- \| --- \| \|  \| **Questions** \| Responses \| Notes \| \|  \| Please enter the name of your trust. \|  \| This is so we can monitor which participating centres have completed this questionnaire. \| \|  \| Please enter the name of your hospital. \|  \| This is so we can monitor which participating centres have completed this questionnaire. \| \|  \| Does your trust have a defined pathway for managing pregnant patients with appendicitis? \| Yes; No \|  \| \|  \| Does your trust have a defined pathway for managing pregnant patients with other intra-abdominal surgical conditions? \| Yes; No \|  \| \|  \| Does your trust have a defined pathway for imaging pregnant patients with acute abdominal pain? \| Yes; No \|  \| \|  \| Are there facilities in place to allow ultrasound scans to be performed to investigate acute abdominal pain in pregnant patients at your site:  Monday-Friday, daytime (08:00-17:00)  Monday-Friday, in the evening (17:00-21:00)  Monday to Friday, overnight (21:00-08:00)  Over the weekend, daytime (08:00-17:00)  Over the weekend, in the evening (17:00-21:00)  Over the weekend, overnight (21:00-08:00) \| Yes; No; In exceptional circumstances  Yes; No; In exceptional circumstances  Yes; No; In exceptional circumstances  Yes; No; In exceptional circumstances  Yes; No; In exceptional circumstances  Yes; No; In exceptional circumstances \| Examples of “exceptional circumstances”: calling in an off-site specialist radiographer to perform the scan, prior arrangement for a specialist radiographer to be present outwith their regular schedule, or discussion/arrangement between patient’s consultant and consultant radiologist, etc.  You may need to consult a radiology colleague to answer this question. \| \|  \| Are there facilities in place to allow MRI scans to be performed to investigate acute abdominal pain in pregnant patients at your site:  Monday-Friday, daytime (08:00-17:00)  Monday-Friday, in the evening (17:00-21:00)  Monday to Friday, overnight (21:00-08:00)  Over the weekend, daytime (08:00-17:00)  Over the weekend, in the evening (17:00-21:00)  Over the weekend, overnight (21:00-08:00) \| Yes; No; In exceptional circumstances  Yes; No; In exceptional circumstances  Yes; No; In exceptional circumstances  Yes; No; In exceptional circumstances  Yes; No; In exceptional circumstances  Yes; No; In exceptional circumstances \| Examples of “exceptional circumstances”: calling in an off-site specialist radiographer to perform the scan, prior arrangement for a specialist radiographer to be present outwith their regular schedule, or discussion/arrangement between patient’s consultant and consultant radiologist, etc.  You may need to consult a radiology colleague to answer this question. \| \|  \| Are there facilities in place to allow CT scans to be performed to investigate acute abdominal pain in pregnant patients at your site:  Monday-Friday, daytime (08:00-17:00)  Monday-Friday, in the evening (17:00-21:00)  Monday to Friday, overnight (21:00-08:00)  Over the weekend, daytime (08:00-17:00)  Over the weekend, in the evening (17:00-21:00)  Over the weekend, overnight (21:00-08:00) \| Yes; No; In exceptional circumstances  Yes; No; In exceptional circumstances  Yes; No; In exceptional circumstances  Yes; No; In exceptional circumstances  Yes; No; In exceptional circumstances  Yes; No; In exceptional circumstances \| Examples of “exceptional circumstances”: calling in an off-site specialist radiographer to perform the scan, prior arrangement for a specialist radiographer to be present outwith their regular schedule, or discussion/arrangement between patient’s consultant and consultant radiologist, etc.  You may need to consult a radiology colleague to answer this question. \| \|  \| Are the obstetrics & gynaecology and general surgery departments based at the same site within your trust? \| Yes; No \|  \| \|  \| Are the general surgery and neonatal services based at the same site within your trust? \| Yes; No \|  \| \|  \| If the general surgery and Obstetrics & Gynaecology/ Neonatal services are based at different sites, which of the following best describes ­usual practice when a pregnant patient requires emergency non-obstetric abdominal surgery (such as appendicectomy or laparotomy), after the age of foetal viability? \| N/A (all services are based at the same site);  The operation is performed at the site where specialist obstetric/neonatal facilities are available as far as possible;  The operation is performed at the site where the General Surgery department is based;  The operation is performed at the site where the General Surgery department is based, with an obstetrics specialist &/or neonatal specialist is readily available. \| Take the age of foetal viability to mean >=22 weeks gestation \| \|  \| Which of the following best describes usual anaesthetic practice in your trust for pregnant patients requiring emergency non-obstetric abdominal surgery (such as appendicectomy or laparotomy)? \| This would be administered by the on-call anaesthetist, regardless of their sub-specialty;  This would be administered by an anaesthetist with experience or expertise in obstetric anaesthesia. \| You may need to consult an anaesthetic colleague to answer this question. \| \|  \| What was/is the standard approach to an appendicectomy for acute appendicitis in the **non-pregnant** patient in your hospital:  in 2013?  In 2018?  Currently (2023)? \| Laparoscopic; Open  Laparoscopic; Open  Laparoscopic; Open \| Please take the “standard” approach to mean the approach that would be adopted as first-line in the majority of patients by the majority of surgeons in your hospital unless (relative) contraindications identified pre-operatively warranted a change in strategy. \| |
| --- | --- | --- | --- | --- | --- | --- | --- | --- | --- | --- | --- | --- | --- | --- | --- | --- | --- | --- | --- | --- | --- | --- | --- | --- | --- | --- | --- | --- | --- | --- | --- | --- | --- | --- | --- | --- | --- | --- | --- | --- | --- | --- | --- | --- | --- | --- | --- | --- | --- | --- | --- | --- | --- | --- | --- | --- | --- | --- | --- | --- |

**Table 2. Retrospective study – data collection template**

|  | **Field** | **Responses** | **Notes** |
| --- | --- | --- | --- |
| *Patient characteristics* | | | |
|  | Study ID |  |  |
|  | Age at initial presentation (years) | 18-25; 26-30; 31-35; 36 and above |  |
|  | Gestational age at presentation (weeks) | [value] weeks; [value] days |  |
|  | Parity | Primiparous; Multiparous |  |
|  | Co-morbidities  Pre-pregnancy BMI  Diabetes or gestational diabetes  Smoker  Immunosuppression  Previous C-section  Hypertension (pre-existing or gestational)  Other cardiovascular comorbidity  Previous presentation with acute appendicitis  ASA | [value]  Y;N  Y;N  Y;N  Y;N  Y;N  Y;N  Y;N  I II III IV V |  |
| *Details of initial presentation and admission with possible appendicitis* | | | |
|  | Date of initial presentation to hospital with suspected appendicitis | dd-mm |  |
|  | Year of initial presentation | 01.10.2013 – 30.09.2017; 01.10.2017 – 30.09.2023 |  |
|  | White cell count (X10^9^/L) at initial hospital presentation | [value] |  |
|  | Neutrophil count (X10^9^/L) at initial hospital presentation | [value] |  |
|  | CRP at initial hospital presentation (mg/L) | [value] |  |
|  | Was the patient already an inpatient at the time of symptom onset? | Yes; No |  |
|  | Which team did the patient initially present to with appendicitis in hospital? | Accident and Emergency; Obstetrics &/or gynaecology; General Surgery; Medicine; Other (specify) | Only if answer to question 11 is No |
|  | Was the patient admitted to hospital at the time of presentation? | Yes; No | Only if answer to question 11 is No |
|  | Date of admission | dd-mm | Only if answer to question 11 is No |
|  | Primary admitting team | Obstetrics &/or gynaecology; General Surgery; Medicine; Other (specify) |  |
|  | Date of review by General Surgery (if not admitted under General Surgery) | dd-mm; N/A (admitted under General Surgery) | Only if answer to question 15 is not General Surgery |
|  | Was the patient’s care transferred to General Surgery? | Yes; No | Only if answer to question 15 is not General Surgery |
|  | Date of transfer to General Surgery (if not admitted under General Surgery) | dd-mm; N/A (admitted under General Surgery) | Only if answer to question 17 is Yes |
| *Details of diagnosis* | | | |
|  | How was the diagnosis of appendicitis first confirmed? | Imaging; Intra-operative findings; Neither of the above - treated as appendicitis on the basis of clinical suspicion | Option 3 refers to instances where the patient was managed as having acute appendicitis even if not proven on imaging or intra-operatively |
|  | Grade of senior-most member of General surgical team making or confirming the diagnosis of appendicitis | SHO or equivalent; SpR or equivalent; Consultant grade |  |
|  | Date of first imaging | dd-mm |  |
|  | Time of first imaging | Daytime (08:00-17:00); Evening (17:01 – 21:00); Night time (21:01-07:59) |  |
|  | Modality of first imaging and findings on first imaging | **Ultrasound**  Appendix appearance: normal/ abnormal /not seen; Surrounding echogenic fat: Yes/No/Not reported;  Free fluid: Yes/No/Not reported;  Focal tenderness on compression: Yes/No/Not reported;  Thickened/oedematous appendix wall: Yes/No/Not reported;  Appendicolith: Yes/No/Not reported;  Dilated appendix: Y/N/Not reported  **MRI**  Appendix visualised: Yes/No  Dilated appendix: Y/N/Not reported Stranding or inflammatory change around appendix or caecum or in right iliac fossa Yes/No/Not reported  Appendicolith: Yes/No/Not reported Perforation: Yes/No/Not reported Abscess or collection: Yes/No/Not reported  **CT**  Appendix visualised: Yes/No  Dilated appendix: Y/N/Not reported Stranding or inflammatory change around appendix or caecum or in right iliac fossa Yes/No/Not reported  Appendicolith: Yes/No/Not reported Perforation Yes/No/Not reported Abscess or collection Yes/No/Not reported | A dilated appendix is defined as one measuring >6mm in diameter  Perforation – this may have been described indirectly without using the term “perforation” e.g. focal defect in appendiceal wall, appendicular abscess, extraluminar gas, extraluminal appendicolith. You may need to consult a surgical colleague to help with interpretation of the report. |
|  | Date of second imaging | N/A (no subsequent imaging); dd-mm |  |
|  | [If applicable]  Time of second imaging | [same values as field no. 22] |  |
|  | [If applicable]  Modality of second imaging and findings on second imaging | [same values as field no. 23] |  |
|  | Date of third imaging | N/A (no subsequent imaging); dd-mm |  |
|  | [If applicable]  Time of third imaging | [same values as field no. 22] |  |
|  | [If applicable]  Modality of third imaging and findings on third imaging | [same values as field no. 23] |  |
| *Details of treatment* | | | |
|  | How was this episode of appendicitis managed? Tick all that apply. | No treatment; Antibiotics; Radiological drain; Surgery |  |
|  | Date of initiation of antibiotics | dd-mm | If answer to Q30 includes “antibiotics”. |
|  | Date of radiological aspiration or drain | dd-mm | If answer to Q30 includes “radiological drain” |
|  | Date of operation for suspected/confirmed appendicitis | dd-mm | If answer to Q30 includes “surgery” |
|  | Time of operation | Daytime (08:00-17:00); Evening (17:01 – 21:00); Night time (21:01-07:59) | If answer to Q30 includes “surgery” |
|  | Anaesthesia | General; Regional | If answer to Q30 includes “surgery” |
|  | Name of operation documented on operation note | Appendicectomy; Right hemicolectomy; Drainage of sepsis and/or washout only; Other – please specify | If answer to Q30 includes “surgery” |
|  | How was the operation performed? | Open via right lower quadrant incision; Open via midline laparotomy; Laparoscopic; Laparoscopic converted to open via right sided incision; Laparoscopic converted to midline laparotomy | If answer to Q30 includes “surgery” |
|  | Operative findings –appendix  Select all that apply. | Normal; Inflamed; Perforated; Gangrenous | If answer to Q30 includes “surgery” |
|  | Operative findings- contamination | None; Localised; generalised  Serous; Pus; Faecal matter | If answer to Q30 includes “surgery” |
|  | Operative findings- associated abscess | Yes; No | If answer to Q30 includes “surgery” |
|  | Histology | Normal; Acute appendicitis; Malignancy; Other – specify [value] |  |
|  | Post-operative antibiotics | Yes; No |  |
|  | Duration of post-operative antibiotics | [value] days | If answer to above is “Yes” |
| *Outcomes* | | | |
|  | Date of discharge | dd-mm |  |
|  | Did the patient experience any complication within 30 days of initial admission (or initial presentation with appendicitis if the patient was already an inpatient at the time of presentation)?  Select all that apply. | None; Wound infection (requiring abx); Wound infection (requiring drainage); intra-abdominal collection (requiring antibiotics); intra-abdominal collection (requiring drain); Ileus; hospital acquired pneumonia; DVT/PE; Return to theatre; Level 2/3 care; Death |  |
|  | 30-day re-attendance with an **acute** presentation – date | dd-mm; N/A (did not reattend within 30 days) | Re-presentation for an acute problem within 30 days of initial admission (or initial presentation with appendicitis if the patient was already an inpatient at the time of presentation). Any planned follow-up such as obstetric scan should be disregarded |
|  | 30-day re-attendance reason (tick all that apply) | Repeat presentation with appendicitis/suspected appendicitis; Complication of appendicectomy; Pregnancy-related; Other – specify [value] |  |
|  | Re-attendance(s) with appendicitis during this pregnancy? | Yes; No |  |
|  | Date of first re-attendance with appendicitis during this pregnancy? | dd-mm | If answer to Q48 is “Yes” |
|  | First re-attendance with appendicitis during this pregnancy – management | Antibiotics; Interventional radiology (drain); Operation – appendicectomy; Operation – other; please specify [value] | If answer to Q48 is “Yes” |
|  | Any further re-attendance(s) with appendicitis during this pregnancy? | Yes; No | If Q48-50 answered |
|  | Date of second re-attendance with appendicitis during this pregnancy? | dd-mm | If Q48-50 answered |
|  | Second re-attendance with appendicitis during this pregnancy – management | Antibiotics; Interventional radiology (drain); Operation – appendicectomy; Operation – other; please specify [value] | If Q48-50 answered |
|  | How did the pregnancy end? | Livebirth; Stillbirth; Neonatal death; Termination of pregnancy (surgical/medical); Miscarriage |  |
|  | Mode of delivery | Vaginal (including assisted deliveries)  C-section | If answer to Q54 is “livebirth” or “stillbirth” |
|  | Date when pregnancy ended | dd-mm |  |
|  | Gestational age when pregnancy ended | [value] weeks; [value] days |  |
|  | Small for gestational age | Y; N | This is defined as a birth weight less than the 10^th^ centile for gestational age. Use this calculator to work this out, by entering foetal sex, gestational age (weeks + days) and weight (kg): http://intergrowth21.ndog.ox.ac.uk/en/ManualEntry |
